# Acral Extragenital Lichen Sclerosus and Its Dermoscopic Findings

**DOI:** 10.7759/cureus.70995

**Published:** 2024-10-07

**Authors:** Francisco Javier Alvarez-Rubio, Víctor Manuel Tarango-Martinez

**Affiliations:** 1 Dermatology, Instituto Dermatológico de Jalisco "Dr. José Barba Rubio", Guadalajara, MEX

**Keywords:** acral extragenital lichen sclerosus, dermatology, dermoscopy, extragenital lichen sclerosus, lichen sclerosus

## Abstract

Lichen sclerosus (LS) is a chronic inflammatory disorder that predominantly affects the genital region of postmenopausal women, often resulting in significant morbidity due to pruritus and pain. Extragenital manifestations are rare and can present a diagnostic challenge. We report a case of a 65-year-old female presenting with white papules and plaques, some with a cribriform appearance, in an acral distribution. We detail the dermoscopic findings observed and provide a review of the relevant published literature on this topic.

## Introduction

Lichen sclerosus (LS) is a chronic inflammatory mucocutaneous disorder, typically presenting as white, atrophic patches or plaques on the vulva, perineum, or prepuce. A less common extragenital form may involve regions such as the upper trunk, neck, shoulders, and wrists. Histopathologically, it is characterized by thinning of the epidermis, basal cell degeneration, band-like lymphocytic infiltrate, and replacement of the normal papillary dermis with homogenized collagen [1, 2]. In its early stages, extragenital LS may resemble other dermatoses, such as morphea, lichen planus, chronic eczema, vitiligo, and flat warts, due to the absence of the hallmark features of sclerosis and atrophy, which become more pronounced as the disease advances [3]. Dermoscopy has emerged as an essential tool for diagnosing a wide range of dermatologic conditions. Herein, we present a case of LS with exclusive extragenital involvement, characterized by an acral distribution and notable dermoscopic findings.

## Case presentation

**Figure 1:**
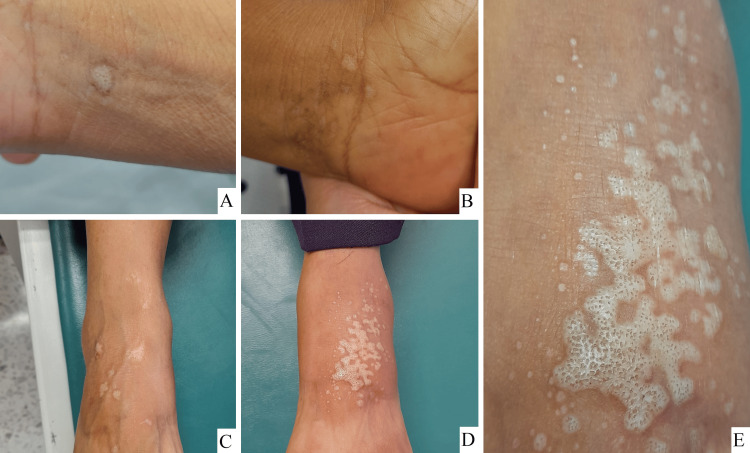
Clinical findings Papules and plaques with comedo-like openings, yellowish and white-colored on the anterior side of right (A) and left (B) wrist, and right (C) and left ankle; close up of left ankle (E).

A 65-year-old woman presented to our clinic with a six-month history of what she described as an 'allergy' in her hands and feet, accompanied by mild pruritus. Physical examination revealed numerous yellowish-white papules and plaques, some with comedo-like openings (cribriform appearance) and mild infiltration, located on the volar aspects of the wrists and anterior ankles (Figure 1). No abnormalities were found on the scalp, mucous membranes, or nails.

**Figure 2:**
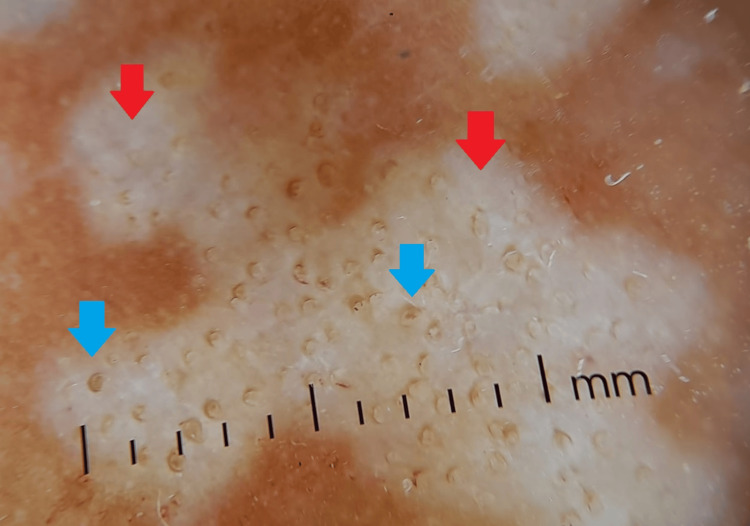
Dermoscopy findings Polarized dermoscopy shows structureless white areas (red arrows) with comedo-like openings (blue arrows).

The differential diagnoses included morphea, acrokeratosis verruciformis of Hopf, lichen planus, and extragenital LS. The dermoscopic evaluation revealed white, structureless areas with comedo-like openings (Figure 2). 

An incisional biopsy was performed, and the histopathological report described an acanthotic epidermis with orthokeratotic hyperkeratosis, follicular plugs, a lichenoid lymphoid infiltrate adjacent to the basal membrane, and homogenized collagen in the papillary dermis, consistent with a diagnosis of extragenital LS. Clobetasol 0.05% cream was initiated twice daily for four weeks, resulting in satisfactory clinical improvement, with no recurrences observed after eight months of follow-up.

## Discussion

Lichen sclerosus typically manifests in the genital region; however, 15% to 20% of cases involve extragenital sites, and only 6% present exclusively in extragenital locations [4]. Although precise prevalence data are lacking, women are more frequently affected than men, with a reported female-to-male ratio ranging from 3:1 to 10:1 [5]. The most commonly involved extragenital areas include the upper trunk and proximal extremities, where asymptomatic opalescent white papules and plaques gradually develop sclerosis and atrophy [6]. In the early stages, comedo-like openings may be observed, although these typically regress over time [3].

Although the exact etiology of LS remains unclear, several contributing factors have been identified, including genetic predisposition (HLA-DQ7), autoimmune conditions (such as autoimmune thyroid disease), infectious agents (e.g., *Borrelia burgdorferi*, Epstein-Barr virus, hepatitis C virus, human papillomavirus), as well as hormonal, local, and traumatic influences (e.g., lack of circumcision in men, friction, humidity, urine exposure). Histologically, early-stage LS is characterized by epidermal acanthosis with posterior atrophy, often accompanied by orthokeratotic hyperkeratosis and follicular plugs, lymphocytic and mononuclear cell infiltrates, and degeneration of the basal layer [2]. Ultrapotent topical corticosteroids are the first-line treatment [7].

Dermoscopy typically reveals white, structureless areas and comedo-like openings (follicular plugs) [8]. Other possible findings include scales, a pseudo-pigment pattern, and chrysalis-like structures. Comedo-like openings are generally seen only in the early stages of extragenital LS (within the first 1.5 years), while chrysalis-type structures tend to appear in more advanced stages [4, 9]. Extragenital LS and morphea can be clinically similar; however, morphea shows white clouds crossed by spreading telangiectasia on dermoscopy [10].

## Conclusions

Dermoscopy is a valuable diagnostic tool for multiple skin conditions, emphasizing the need to further expand knowledge in this field. A deeper understanding of dermoscopic features across various dermatologic pathologies can significantly enhance diagnostic accuracy. In cases of extragenital LS, dermoscopy offers crucial insights by revealing characteristic findings, such as white structureless areas and comedo-like openings. 
